# Atypical Antipsychotics and Other Therapeutic Options for Treatment of Resistant Major Depressive Disorder

**DOI:** 10.3390/ph3123522

**Published:** 2010-12-03

**Authors:** Rubo J. Seo, Holly MacPherson, Allan H. Young

**Affiliations:** Institute of Mental Health, Department of Psychiatry, University of British Columbia, 5950 University Blvd. Vancouver, BC V6T 1Z3, Canada; E-Mails: donyseo@hotmail.com (R.J.S.); hollymacpherson@gmail.com (H.M.);

**Keywords:** treatment resistant disorder, major depressive disorder, augmentation, atypical antipsychotic, typical antipsychotic, antidepressant, selective serotonin reuptake inhibitor, electroconvulsive therapy

## Abstract

Antidepressant therapies, such as selective serotonin reuptake inhibitors (SSRIs), are current first-line treatments for Major Depressive Disorder. However, over 50% of treated patients show an inadequate response to initial antidepressant therapy. If the therapeutic outcomes from two antidepressant therapies are suboptimal, potentially resulting in Treatment Resistant Depression, subsequent strategies include switching to another antidepressant or augmenting treatment by combining with other agents. When combined with SSRIs, atypical antipsychotics have supplementary action on dopaminergic and noradrenergic systems. Studies on combined treatment with atypical antipsychotics have shown significantly increased remission rates, shortened response times, and favorable side effects. Augmentation of antidepressants with atypical antipsychotics is now an acceptable treatment strategy which leads to increased remission rates and better outcomes for patients.

## 1. Introduction

Major Depressive Disorder (MDD) is the third leading cause of disease burden worldwide [1] and will affect between 13-16% of people during the course of their lifetime [2,3]. Characterized by symptoms which include low mood, along with low self-esteem, and/or loss of interest or pleasure in normally enjoyable activities [4], the impact of depression can be severe. It causes significant impairment, poor social and occupational functioning, and is associated with high rates of psychiatric comorbidity, increased rates of physical health problems and raised mortality. Of those hospitalized with severe depression, approximately 16% eventually commit suicide [5]. Episodes of major depression can occur within the context of major depressive disorder or bipolar disorder [4] and treatment options will not necessarily be the same for both. However, this review focuses on depression as part of MDD.

### 1.1. Potential Treatments for MDD

Antidepressant medications have been in use for the treatment of MDD since the 1950s. Tricyclic antidepressants (TCAs) and monoamine oxidase inhibitors (MAOIs) were the first modern pharmacotherapies used for depression but have been less used lately because they are associated with problematic side effects and drug interactions; TCAs are associated with anticholinergic (dry mouth, blurred vision, constipation), antihistaminergic (sedation, carbohydrate craving, increased appetite), and anti-α-1-adrenergic (orthostatic hypotension) side effects, whereas MAOIs are associated with insomnia, sedation, and orthostatic hypotension [6]. Derivative compounds such as norepinephrine reuptake inhibitors (NERIs) and the serotonin-norepinephrine reuptake inhibitors (SNRIs) as well as other selective antidepressants like the noradrenaline-dopamine reuptake inhibitors (NDRIs; bupropion) and tetracyclic antidepressants (TeCA; mirtazapine) were introduced from the 1970s onwards and are believed to be more tolerable than TCAs and MAOIs. In particular, serotonin reuptake inhibitors (SSRIs) are believed to have good tolerability profiles and accounted for more than half of all antidepressant prescriptions in 2006 [7,8]. The naturally occurring molecule, *S*-adenosyl-L-methionine (SAMe), has also been used as an alternate pharmacologic approach and has shown antidepressant efficacy comparable to TCAs with minimal side effect profiles. In addition, psychosocial therapy should be added to ongoing pharmacotherapy to maximize improvement in remission of symptoms as well as facilitating patients to regain normal everyday functioning.

### 1.2. Treatment Resistant Depression

Despite the plethora of available antidepressants with differing modes of action, only modest differences in efficacy have been found. According to a Sequenced Treatment Alternatives to Relieve Depression (STAR*D) report, only one-third of patients receiving initial antidepressant therapy achieve remission [9]. It has been found that 29-46% of depressed patients on SSRIs remain unresponsive to treatment [10]. In addition, the STAR*D study also reported that after each failed pharmacotherapy trial, remission rates decline; after two or three failed treatment trials, subsequent remission rates averaged less than 15% [9].

Patients who have partial or no response to pharmacotherapy may be characterized as having treatment resistant depression (TRD). Numerous definitions and stages of treatment resistance have been suggested. TRD has been described clinically along a continuum, from first episode patients failing single antidepressant treatments to chronic patients failing multiple combined and/or augmented therapies [11]. Thase and Rush have proposed five stages of TRD, varying from an unsuccessful trial of one antidepressant with adequate dose and duration (Stage I) to failure of three to four classes of antidepressants plus ECT (Stage V) [12]. General consensus defines TRD as failure to respond to two different prospective trials of antidepressants of adequate dose and duration.

The clinician’s initial response to a potential case of TRD should include a re-evaluation of the diagnosis, an examination for psychiatric comorbidities that may hinder therapeutic response, and verification that the patient is complying with treatment. For those suffering from treatment resistant depression, in which the therapeutic response to treatment has plateaued or has remained unchanged within 3 weeks, the first step for many would be to increase the dose of the initial SSRI. If there is no significant response to the dose increase within 2-4 weeks, a clinician should consider switching to another SSRI or to another pharmacological class of antidepressant. Switching pharmacotherapy is suitable for those patients who either show no response to initial treatment, show only an inadequate response to initial treatment even after an increase in dose, or their response was outweighed by side effects. For the partial responder whose pharmacotherapy has not been restricted by side effects, augmentation, *i.e.*, the utilization of adjunctive medication to enhance the action of the initial antidepressant medication is particularly useful. Pharmacological profiles of the augmentation agent must be carefully accounted for as it may aggravate existing side effects through, for instance, drug-drug interactions. Thus, clinicians must choose the augmentation agent with caution and use of symptom characteristics (both illness and side effects) might be used as a guide [13].

### 1.3. Pharmacologic Profile of Depression

The symptoms that characterize MDD are presumed to be associated with brain monoaminergic neuronal dysfunction. The role of serotonin and norepinephrine in the actions of antidepressants is well documented. Yet, because many depressed patients do not respond to SSRIs, NERIs, or combined agents, it is likely that other biochemical factors are involved. Recently, deficits in dopaminergic activity have been found in patients with TRD [14], suggesting that the pathogenesis of this variant of depression may reflect disturbances in all three neurotransmitter systems [15]. A large number of other neurotransmitters, from glutamate to neuropeptides, may be involved but are beyond the limited scope of the review.

## 2. Augmentation Therapy

All the published studies report that many patients with MDD commonly fail the first line antidepressant treatment. The general concensus approach to such cases are 2^nd^, 3^rd^, and even 4^th^ line of treatment in an attempt to achieve full remission of symptoms [16]. Considering that approximately 70% of patients do not remit through an adequate course of first antidepressant treatment [9,17,18], alternatives such as adding an effective second agent with improved safety and tolerability profiles to an ongoing antidepressant medication, referred to as augmentation therapy, can avoid patients from unnecessary switching between antidepressants which may jeopardize the remission rates and even aggravate existing side effects as suggested in the STAR*D study [9].

### 2.1. Augmentation Agents

Augmentation therapy is generally recommended to patients who have shown either treatment-resistant or insufficient response to an antidepressant regimen. The possible augmenting agents, which include lithium, triidothyronine (T3), benzodiazepines, *S*-adenosyl-L-methionine carbamazpine, valproic acid, buspirone, psychostimulants, typical antipsychotics and atypical antipsychotics, have been reported to improve clinical efficacy by treating residual symptoms [19,20] and broaden the treatment’s neurochemical profile by adding complementary pharmacodynamic action [21,22]. A common trial design is for the depressed patient to be first treated with an open-label antidepressant monotherapy for 6 to 8 weeks. Patients who show inadequate response are then randomized either to the second agent of interest or placebo [16]. According to a meta-analysis of 10 randomized-controlled trials, remission rates for the augmentation group were significantly superior when compared to the placebo groups (p < 0.0001) [23]. However, possible adverse effects involved with combining two medications must be considered when choosing for the right augmentation agent as it may potentiate already existing adverse effects, or alter the drug levels in plasma concentration. For instance, it is now generally understood that the transport of drugs from the intestine to the blood and from the periphery to the brain is facilitated by not only passive diffusional processes but also via the membrane protein P-glycoprotein (P450), a member of the adenosine triphosphate-binding cassette superfamily of transport-proteins, which transports antipsychotics such as risperidone through the gastrointestinal tract and the blood brain barrier. Depending on the nature of such substrates, it may induce or inhibit the action of P450 and likely to increase or decrease, respectively, the transport of the antidepressant drugs into the brain. [24]. Physicians must consider this notion in order to maintain the plasma drug levels in the therapeutically optimal range.

### 2.2. Lithium Augmentation

Lithium augmentation of patients with treatment resistant major depressive disorder was based on preclinical data that showed a positive association between lithium levels and presynaptic formation, storage, and release of serotonin in rats. Lithium augmentation, which is postulated to synergistically increase serotonergic action of antidepressants on brain 5-HT pathways [25], was first reported to augment patients with partial or absent response to T3 in 1981 and subsequently became a gold standard to treat patients with treatment resistant major depressive illness [26,27]. As one of the best documented approaches in TRD patients with existing TCA treatment, lithium augmentation has shown antidepressant efficacy and relative safety in both open and controlled studies [26,28,29]. The safety profiles of lithium augmentation in ongoing TCA therapy was supported by Schweitzer *et al.*’s work, showing that there were no serious or accentuation of adverse effects due to combination of two agents: anticholinergic and cardiovascular complications, nausea, diarrhea, abdominal pain, muscle weakness, and fine tremor from the lithium augmentation [30]. Also, no serious adverse effects were reported concerning lithium augmentation of MAOI therapy. For instance, reported risks for hypertensive crises due to the lithium augmentation were not higher than when MAOIs were used as a monotherapy. Further double-blind, randomized, placebo-controlled trials showed that fluoxetine and citalopram combination with lithium showed superior response without any indications of accentuation or aggravation of adverse effects compared to placebo [31,32]. However, caution must be present when augmenting SSRIs with lithium for both agents increase serotonin neurotransmission *via* different mechanisms and may potentiate serotonin syndrome marked by confusion, restlessness, myoclonus, hyperreflexia, diaphoresis, shivering, tremor, hypomania, diarrhea, and possibly death [33]. There have been two reported cases that therapeutical interactions between lithium and fluoxetine resulted in mania and hypomania [34].

### 2.3. Triiodothyronine (T3) Augmentation

Adjunctive T3 therapy is one of the most widely studied augmentation methods [20]. A direct comparison between lithium and triiodothyronine (T3) augmentation of TCA, indicated both to be equivalently effective and more effective than placebo [35]. While no significant differences were reported between these agents, the group augmented with T3 showed tendencies to greater numbers of responders and remitters, greater decreases in Hamilton Rating Scale for Depression (HAM-D) and Quick Inventory of Depressive Symptomatology (Self Report; QIDS-SR) scores, and greater tolerability to treatment. T3 not only may hold a slight advantage over lithium in efficacy and tolerability but also is easier to use and there is no need for blood monitoring [20]. Stein *et al.* supports the advantage of T3 over lithium augmentation of imipramine by showing enhanced and accelerated recovery with no association with aggravations of the attendant adverse effects [36]. The possible common adverse effects (drowsiness, dizziness, dry mouth, tremor, anxiety, cardiotoxicity, agitation, and insomnia) were all found to be mild or absent [37]. T3 augmentation of patients with treatment resistant major depressive disorder to phenelzine (MAOIs) also showed clinical improvement with no significant adverse effects [38,39]. 

### 2.4. Benzodiazepine Augmentation

According to a recent Cochrane Review, benzodiazepine augmentation with antidepressants may be beneficial for the treatment of depression. It was found that compared to antidepressant treatment alone, adjunctive benzodiazepines decrease drop outs from treatment as well as increasing short-term response up to four weeks. However, these potential benefits should be weighed against possible complications related to benzodiazepine augmentation, such as the development of dependence, a decline in the drug’s effect over time, as well as an increase in “accident proneness” [40].

### 2.5. S-Adenosyl-L-Methionine (SAMe) Augmentation

SAMe is a naturally occurring molecule and has been commercially available in Europe since the late 1970s and as an over-the-counter dietary supplement in the United States since 1999. It has shown an antidepressant efficacy that is superior to placebo and equivalent to the effects of TCAs in a meta-analysis of randomized, double-blind, placebo- or TCA-controlled monotherapy trials [6]. In addition, a positive correlation between the increase of SAMe levels in the cerebrospinal fluid and the improvement in depressive symptoms have been observed [41]. Moreover, an examination of SAMe as an augmentation treatment of SSRIs in patients with treatment resistant major depressive disorder supported its potential efficacy, tolerability and safety [42]. According to the results, SAMe-treated patients and placebo-treated patients showed respective response rates of 36.1% and 17.6%, remission rates of 25.8% and 11.7%, and discontinuation rates due to intolerance of 5.1% and 8.8%, respectively. There was no significant difference in adverse events between these two groups. The suggestive, yet preliminary, evidence of SAMe as an effective antidepressant in patients with treatment resistant major depressive disorder warrants confirmation in future studies so that SAMe might be recommended as a novel augmentation agent to the antidepressant therapy armamentarium. 

### 2.6. Other Types of Augmentation

There have been a few case studies that showed marked improvement in patients with TRD after the addition of carbamazepine to TCAs [43], valproic acid to SSRIs [44] and buspirone to SSRIs [45,46] All the adverse effects reported in these studies were minimal or mild. However, augmenting with these agents (carbamazepine, valproic acid, and buspirone) carries the caveat that they are P450 inducers, inhibitors, and partial 5-HT_1A_ agonists, repectively. Since these agents lack a body of supporting evidence for their use in TRD, physicians must carefully choose these methods as carbamazepine may lower the TCA levels whereas valproic acid may increase the serotonin levels in the brain. Buspirone may potentiate the SSRI side effects such as sexual dysfunction [45,46]. Overall, there were no discrepancies reported for these agents in terms of effectiveness and tolerability between genders; however, the usefulness of such agents in the elderly may be limited by dosing and pharmacological issues [47]. Psychostimulants may pose potential treatment for TRD as examined in case reports and open-label studies [48,49]; however, no double-blind, placebo-controlled trials have been conducted [50]. 

### 2.7. Typical Antipsychotic Augmentation

Typical antipsychotics, such as haloperidol and chlorpromazine, have had a long history in depression treatment. Use of these conventional antipsychotics, however, have waned due to their association with increased risk of extrapyramidal symptoms (EPS) including parkinsonism, dystonia, akathisia, and tardive dyskinesia [51], as well as torsades des pointes and sudden cardiac death [52]. Increasingly, augmentation strategies with atypical antipsychotics, which have lower reported incidences of EPS than typical agents, have gained prominence for the treatment of TRD. Dopamine D_2_ receptors, the principal brain target of antipsychotics, have been found to be involved in eliciting EPS. Typical antipsychotics have a tighter binding affinity at D_2_ receptor than dopamine itself and have a lower dissociation time course than dopamine. Conversely, atypical agents have a weaker binding affinity at D_2_ receptors and have a higher dissociation time course than dopamine. Thus, atypical agents transiently occupy the D_2_ receptors and then rapidly dissociate, allowing for normal dopamine neurotransmission. For effective antipsychotic action, the occupancy is reported to be approximately 65% at D_2_ receptor. However, EPS induction occurs when occupancy of D_2_ receptors by an antipsychotic is greater than 80% [53].

## 3. Atypical Antipsychotic Augmentation Treatments

For treatment-resistant patients, evidence suggests that augmentation treatment with atypical antipsychotics may have better treatment efficacy, safety and tolerability profiles than some other commonly used TRD treatments. Randomized controlled trials, although as yet limited, have failed to demonstrate clear cut evidence for one atypical agent over another [11,54,55,56,57].

### 3.1. Aripiprazole Augmentation

The use of aripiprazole may be efficacious in treating depressive symptoms in patients with schizophrenia [58] and bipolar disorder [59]. Aripiprazole’s pharmacological action may be due to its partial agonist activity at the D_2_, D_3_ and 5-HT_1A_ receptors as well as its antagonistic effect at 5-HT_2A_ receptors [60,61,62,63,64,65].

Augmentation of SSRIs with aripiprazole has been found to be efficacious for the treatment of TRD in multiple randomized, double-blind placebo-controlled studies [60,61,66,67]. Aripiprazole augmentation has shown rapid improvements in Montgomery-Åsberg Depression Rating Scale (MADRS) total scores, as early as the first and second week of treatment with continued improvement throughout the duration of treatment. Aripiprazole augmentation has produced significantly greater remission rates and response rates compared to placebo at the study endpoint, typically 8 weeks. Berman *et al.* showed a 2-fold greater likelihood of remission with adjunctive aripiprazole compared to placebo [60,66].

Adjunctive aripiprazole has been fairly well tolerated; completion rates with aripiprazole were high and discontinuations due to adverse effects were low, similar to those reported for placebo groups. The most common adverse events associated with aripiprazole included akathisia, headache, fatigue, insomnia, tremor, constipation, and restlessness. Aripiprazole was associated with greater weight gain than placebo though findings were not consistent; two studies showed a significant increase in mean weight gain with adjunctive aripiprazole than with adjunctive placebo (+ 2.01 ± 0.17 kg *vs.* + 0.34 ± 0.18 kg; p < 0.001 [53]; + 1.47 ± 0.16 kg *vs.* 0.42 ± 0.17 kg; p < 0.001; LOCF [61]), whereas Berman *et al.* found no significant difference in weight gain between adjunctive aripiprazole and adjunctive placebo (+ 1.2 kg *vs.* + 0.8 kg; p = 0.14; LOCF) [66]. No adverse effects on prolactin levels were found suggesting that aripiprazole augmentation does not worsen the potential for hyperprolactinemia [61,68]. In addition, improvements in sexual function were significantly greater with adjunctive aripiprazole than adjunctive placebo. 

Effects on clinical outcomes were significantly improved after adjunctive aripiprazole, as measured using the Sheehan Disability Scale [61]; significant improvement was found on family and social life items but not work items. These improvements with aripiprazole augmentation may be important given the negative impact residual symptoms have on psychosocial function [69,70]. It has been reported that reaching full symptomatic remission results in greater improvements in functioning than reaching response without remission or non-response. Full remission is also associated with improved prognosis and enhanced psychosocial functioning and quality of life [71]. Given the multiple replications of improved response rates and remission rates in the adjunctive aripiprazole trials as well as well-tolerated safety profile, aripiprazole is well supported as an efficacious augmentation agent for TRD. Due to its efficacy and tolerability, aripiprazole became the first atypical antipsychotic to receive U.S. Food and Drug Administration approval for adjunctive treatment of major depressive disorder in 2008 [51] although it is not clear if this approval will be replicated in every other country.

### 3.2. Clozapine Augmentation

Clozapine, has been found to be efficacious in the treatment of refractory schizophrenia, causes less EPS or tardive dyskinesia than typical antipsychotics and does not elevate prolactin levels [72,73,74]. It has an affinity for dopamine and serotonin receptors [75], which suggests antidepressant potential. Clozapine has shown to be effective in reducing suicidality and suicide attempts in a cohort of neuroleptic-resistant schizophrenic patients [76]; however Sernyak *et al.* did not find any significant differences in the rate of suicide or accidental death in a large cohort study [77]. Clozapine has been found to have a rare but significant risk of causing agranulocytosis, occurring in 1-2% of patients who take this particular atypical agent. Clozapine selectively affects precursors of polymorphonuclear leukocytes in bone marrow and has other haematological effects such as leucopenia, neutropenia and eosinophilia. In a meta-analysis comparing clozapine to other atypical antipsychotics for schizophrenia, clozapine showed a tendency for more nausea, fatigue, hypersalivation, and orthostatic dizziness than the other atypical agents. Given the severity of the adverse events associated with clozapine, its use has been limited only to patients who are resistant to or intolerant of other antipsychotic drugs [78].

### 3.3. Olanzapine Augmentation

Preclinical evidence of the atypical antipsychotic, olanzapine, in combination with the SSRI fluoxetine, increases serotonin, norepinephrine, and dopamine in the rat brain [79]. When a combination of olanzapine and fluoxetine was administered, norepinephrine and dopamine levels increased to 242% and 315% of baseline values, respectively, values significantly higher than either agent alone. These increases in prefrontal norepinephrine and dopamine levels suggest a plausible neurochemical basis for the synergistic antidepressant effect observed in the clinical trials. 

Acute and long-term, double-blind studies of olanzapine alone as well as in combination with fluoxetine have shown mixed results for the treatment of TRD. Thase *et al.* conducted two parallel, 8-week double-blind studies comparing olanzapine monotherapy, fluoxetine monotherapy, and olanzapine/fluoxetine combination [57]. The first study did not find a statistically significant difference in remission rates and response rates between the three treatment options at endpoint; the second study revealed that olanzapine/fluoxetine combination showed significantly greater improvement in efficacy than the other groups. When both studies were pooled, a significant difference in favor of the olanzapine/fluoxetine combination over either agent alone was revealed. Shelton *et al.* also found superior efficacy for the combination of olanzapine/fluoxetine compared to both monotherapies [54], whereas, a further Shelton *et al.* study and that of Corya *et al.* did not find a significant difference between therapies at the endpoint of treatment [11,80]. In these studies, an early and significant improvement in depressive symptoms was shown in the combination groups compared to the monotherapies as early as week 1 and maintained symptom improvement throughout the course of treatment. The monotherapy groups, on the other hand, revealed a steady improvement over the8-week trial, and ultimately had similar improvement to the combination group at the endpoint. These studies found that the time to response and remission was significantly shorter for olanzapine/fluoxetine combination treatment than for fluoxetine and olanzapine monotherapies, suggesting a more rapid onset of action. In a long-term, open-label study Corya *et al.* examined the use of olanzapine/fluoxetine combination over a course of 76-weeks and found high response rates (53%) and remission rates (44%), and low relapse rates (25%) at endpoint [81].

Olanzapine and fluoxetine, as well as the olanzapine/fluoxetine combination treatments were relatively well tolerated. The most frequently reported significant adverse events reported in these studies were somnolence, increased appetite, asthenia, weight gain, headache, dry mouth, nausea, anxiety, tremors, and nervousness [11,54,80]. Findings on significant differences between treatments with olanzapine, fluoxetine, and the combination of both agents for treatment emergent adverse events were again, mixed, but reported as mild to moderate. A significant difference among treatment groups in rates of patient discontinuation due to adverse events was found [57,80]. Fewer patients in the fluoxetine monotherapy group discontinued due to adverse events than in the olanzapine and combination groups. The other studies found similar rates of discontinuation due to an adverse event among all treatment groups [11,54]. A significantly greater weight gain was observed for those treated with olanzapine (in combination as well as alone) than fluoxetine [11,54,57,80,81]. There were no significant differences in changes in vital signs, laboratory analyses, or EPS in any of the studies with olanzapine. Olanazapine/fluoxetine combination as well as fluoxetine monotherapy showed a statistically significant increase in QTc intervals (cardiac function) than olanzapine alone [16]. Similar to the aripiprazole findings, olanzapine/fluoxetine combination treatment was found to have a significantly greater improvement on the leisure and family items of the Sheehan Disability Scales [57].

In both acute and long-term studies of TRD patients, olanzapine/fluoxetine combination therapy demonstrated a rapid, robust and sustained antidepressant effect and a comparable safety profile to that of its constituent monotherapies. At endpoint, olanzapine/fluoxetine combination did not differ significantly from olanzapine and fluoxetine monotherapies; however, the acute onset of improvement with olanzapine/fluoxetine treatment is likely to have influence on clinical treatment strategies. For instance, the speed of antidepressant effectiveness can be an important factor in treatment choice, particularly when it comes to patients with suicidal ideation [11,54,81].

### 3.4. Quetiapine Augmentation

Quetiapine is approved by the United States Food and Drug Administration (FDA) for schizophrenia, MDD, and bipolar associated manic episodes and depression [82]. The pharmacological profiles of quetiapine are similar to that of the first atypical antipsychotic, clozapine, which antagonizes wide range of receptors including serotonergic (5-HT_1A_ and 5-HT_2A_), dopaminergic (D_1_ and D_2_), histaminergic (H_1_), and adrenergic (α1 and α2) receptors [83]. In addition, one of the principal active quetiapine metabolites, norquetiapine, was recently reported to block the human norepinephrine transporter (NET), inhibit norepinephrine reuptake, and exhibit high binding affinity to 5-HT_1A_, H_1_, and α1 receptors [84]. The norepinephrine transporter blockade is a common pharmacologic pathway shared among antidepressants. Also, quetiapine has been reported to have low risk for anticholinergic side effects and low potential for EPS [82]. 

In a randomized, single-blind, placebo-controlled study, patients with MDD and associated anxiety were assigned to either treatment with paroxetine and placebo or paroxetine and quetiapine. After eight weeks, the authors reported significantly decreased HAM-D scores in the combined therapy group compared to the placebo group (P < 0.008). In terms of anxiety, Hamilton Rating Scale for Anxiety (HAM-A) scores showed a significant improvement with quetiapine (P < 0.008) [85].

Another randomized, double-blind, placebo-controlled study investigated the treatment of MDD and the maintenance of remission of symptoms in 72 patients with MDD. The study compared four groups: paroxetine monotherapy, venlafaxine monotherapy, paroxetine and quetiapine combined-therapy, and venlafaxine and quetiapine combined-therapy. Overall, improvement in depressive symptoms and the development of remission occurred in the highest frequency in the paroxetine and quetiapine group followed by venlafaxine and quetiapine group, paraxetine monotherapy, and venlafaxine monotherapy in the order of decreasing improvement in depressive symptoms and the development of remission. [86].

In a 6 week, randomized, double-blind, placebo-controlled study, quetiapine at two different doses (150 and 300 mg/day), was augmented in patients with major depressive disorder. Quetiapine augmentation groups at both doses demonstrated a significant improvement in depressive symptoms as quantified by Montgomery-Åsberg Depression Rating Scale (MADRS) and HAM-D total scores. The significant improvement in MADRS was noted as early as week 1 for both quetiapine doses [87]. In contrast to this finding, El-Khalili *et al.* in their 8-week, multicenter, double-blind, randomized, parallel-group, placebo-controlled study showed significant symptom improvement (MADRS) in week 1 and onward only for the 300 mg/day quetiapine augmentation group; the 150 mg/day quetiapine augmentation group only showed transient efficacy during week 1 [88]. The disagreement in results between the above two studies may be due to the severity of recurrent MDD: 80.6-82.0% and 90.4-94.4%, respectively. It is evident to say that 300 mg/day quetiapine augmentation may be more effective than 150 mg/day in patients with treatment resistant major depressive disorder and a high percentage of recurrent MDD. Additionally, Bauer *et al.* showed a positive association between quetiapine and sleep disturbance; patients who received quetiapine augmentation to ongoing antidepressant treatments demonstrated a beneficial effect on sleep as measured by the HAM-D sleep disturbance item and Pittsburgh Sleep Quality Index (PSQI) scores [87]. 

Gedge *et al.* further supported the effect of quetiapine on the alteration in sleep architecture in patients with treatment resistant major depressive disorder [89]. The study rationale was that 90% of patients with either MDD or bipolar disorder often experience poor sleep quality and quantity [90]. Acute quetiapine treatment (2-4 days) significantly improved sleep architecture involved in total stage 2 sleep, total non-rapid eye movement (non-REM) sleep and the percentage of total sleep time in non-REM sleep. However, the longer-term (21-28 days) effect of quetiapine reverted back to the baseline levels. In terms of depressive symptoms, MADRS total scores significantly improved for both acute and longer-term effect of treatment. Since depression may be associated with decreases in H_1_ binding [91], the antagonism profile of quetiapine at H_1_ receptors is likely to complementarily decrease the depressive symptoms via the inhibition of histamine synthesis [89]. 

Along with sedation, the most commonly reported adverse effects of quetiapine augmentation are somnolence and lethargy in mild to moderate intensity, which is often transient in nature. These adverse effects are presumably due to its strong binding affinity to H_1_ receptors, followed by subsequent down-regulation of histamine synthesis. The transient nature of these side effects implicates rapid tolerability to the actions of quetiapine at H_1_ receptor [92]. Tolerance to somnolence, sedation, and lethargy has been clinically observed, and the H_1_ receptor associated adverse effects decrease over time [93]. Thus, patients must be counseled about the likely transient nature of such adverse effects to facilitate the adherence to the quetiapine regimen.

### 3.5. Risperidone Augmentation

Risperidone, developed from the typical antipsychotic, haloperidol, has binding affinity to serotonin (5-HT_2_) and dopamine (D_2_) receptors in the brain [94]. According to Lakoski *et al.*, the antagonist action of risperidone at 5-HT_2_ receptors amplifies the serotonergic action of SSRIs in the5-HT_2_ receptors [95]. In contrast to conventional antipsychotics, which have the low 5-HT_2_ to high D_2_ ratio profiles [96], risperidone, particularly at low doses, is about 1,000 times more potent in antagonizing 5-HT_2_ receptors than D_2_ receptors [97]. Since greater than 80% occupancy of D_2_ receptor elicits increasing rates of EPS and tardive dyskinesia, augmentation treatment with low dose of risperidone is more favorable with regards to its safety profile [53]. In addition, numerous studies have suggested risperidone as a first-line augmentation agent for it is comparatively non-sedating, not associated with either weight gain or diabetes, and glucose dysregulation [98,99].

In a double-blind, crossover study, risperidone augmentation treatment showed a significant decline in HAM-D (p = 0.04) and Beck Depression Inventory (BDI) (p = 0.02) ratings. Significant improvement in REM sleep, stage 2 sleep, and sleep wakefulness, which was positively associated with the decrease in HAM-D (p = 0.02) ratings, was reported in the risperidone augmentation group as early as two weeks. These data support the antagonist profile of risperidone involved with potentiation of serotonin and norepinephrine neurotransmission through 5-HT_2_ and D_2_ receptors blockade, respectively. It is evident to say that the pharmacologic mechanism of risperidone via facilitation of norepinephrine and serotonin neurotransmission lowers REM sleep, which subsequently correlates to improvement in depression [100].

In a double-blind, placebo-controlled study, augmentation with risperidone showed significant improvement in depressive symptoms [101]. Patients who showed an inadequate response to citalopram monotherapy entered an augmentation treatment with risperidone. Those who responded further entered to the longer-term continuation phase either to risperidone or placebo group. It is noteworthy that non-responders to the original citalopram monotherapy showed marked improvement in symptom reduction during the open-label risperidone augmentation phase and significant delay in relapse rates after the double-blind risperidone continuation (97 days) than placebo continuation (54 days) [55]. 

Numerous open-label and case reports also have supported the benefits of risperidone augmentation of SSRIs at low dose. Patients who showed suboptimal responses to at least two or more SSRI trials showed acute improvement in mood and anxiety symptom reduction, sleep quality, impulse control, sexual dysfunction, agitation, and response rates and remission rates in as little as 1 week of low dose risperidone augmentation treatment [102,103,104,105,106,107].

In summary, the 5-HT_2_ antagonist properties of risperidone in the presence of SSRIs augment the extracellular levels of monoamines. This is likely to be why atypical antipsychotics, such as risperidone, are beneficial in patients with treatment resistant major depressive disorders. The published studies suggest that there are short-term benefits when patients with TRD are augmented with risperidone. However, benefits from the longer-term continuation of risperidone may be limited to a subgroup of patients who were fully unresponsive to the original antidepressant monotherapy.

### 3.6. Ziprasidone Augmentation

Ziprasidone was approved by FDA in 2001 as treatment in schizophrenia and mania or mixed states associated with bipolar disorder. Besides its binding affinity for the same receptors as many other atypical antipsychotics including dopaminergic (D_2_), serotonergic (5-HT_2A_, 5-HT_2C_, and 5-HT_1D_) receptors, ziprasidone has a singular pharmacological property as a strong 5-HT_1A_ receptor agonist [108,109]. Preclinical studies have suggested that the strong 5-HT_1A_ agonist properties of ziprasidone are associated with release of dopamine in rat prefrontal cortex and inhibition of neuronal uptake of serotonin, norepinephrine, and dopamine [109,110,111]. Such a receptor binding profile has suggested that ziprasidone may be useful in treating patients with treatment resistant major depressive disorder in conjunction with the ongoing antidepressant regimen. In terms of common adverse effects shared among atypical antipsychotics, ziprasidone is less likely to cause weight gain, elevation in prolactin levels, and EPS than other atypical antipsychotics. Even though FDA labels the use of ziprasidone with a warning due to possible mortality in the elderly with dementia related psychosis and potential for QTc prologation, clinical studies showed a small effect of ziprasidone on the QTc interval between 6 to 10ms, no serious electrocardiographic changes, no severe cardiac adverse events, or tardive dyskinesia [112].

Papakostas *et al.* demonstrated the potential efficacy of ziprasidone augmentation in patients with treatment resistant major depressive disorder [56]. Twenty patients were enrolled in this open-label trial and experienced 50% and 25% response and remission rates, respectively. In addition, data showed considerable improvement after week 1 of treatment. The acute response may be due to dopamine accumulation in the prefrontal cortex. In terms of safety and tolerability profiles, no patient reported any adverse effects or demonstrated a significant increase in QTc. However, 20% of patients dropped out due to intolerability. In summary, data from ziprasidone augmentation with conventional antidepressants warrants further controlled clinical trials in patients with treatment resistant major depressive disorder [56].

## 4. Electroconvulsive Therapy

If a treatment resistant patient does not show an adequate response to several augmentation treatments, electroconvulsive therapy (ECT) may be advantageous. ECT has long been used as an effective treatment for depression. There are 100,000 patients in the US alone who receive ECT each year, and one million worldwide. In a randomized control trial of patients treated with ECT for depression (both as part of major depression and also bipolar disorder), Kellner *et al.*, found the overall remission rate of 55% with ECT in the intention to treat analysis [113]. Despite its effectiveness against depression, the use of ECT remains controversial and is not fully trusted by some practitioners and patients [114]. However, according to Semkovska and McLoughlin, in their meta-analysis of84 studies (n = 2981), most cognitive variables showed small to medium improvements six months after ECT [115]. What is noteworthy is that all the impaired cognitive domains (cognitive status screening, processing speed, attention/working memory, verbal episodic memory, visual episodic memory, spatial problem solving, executive function and intellectual ability) found in the subacute period (0-3 days) improved after six months and none deteriorated. The subacute transient cognitive impairment among remitters after ECT might be attributable to a sudden modulatory change of brain function impacting on cognition function, which temporarily outweighs the changes associated with the improved depressive symptoms in the subacute period [116]. Considering that there is a positive association of ECT with cognitive improvement in patients with ECT-treated remitted depression, ECT may be clinically favorable for patients with treatment resistant major depressive disorder who have failed augmentation therapies with an atypical antipsychotic.

## 5. Conclusions

The impact of major depressive disorder is severe. The total cost of depression in the British adult population was estimated to be in excess of UK£9 billion in 2000. Cost-effectiveness of pharmacotherapy for depression is affected by dosage practice as well as tailoring of adequate medications. Inadequate medications and dosing reduce effectiveness, which likely impact societal burden due to of working days lost, poor performance and quality of life [117].

Many patients fail their first-line treatments and carry residual symptoms, which is a strong indicator of depression relapse. In order to treat the residual symptoms, older adjunctive methods to first-line antidepressants, including lithium, T3, and typical antipsychotics, have been used. Atypical agents have better pharmacological profiles for the treatment of depression than traditional agents, and have repeatedly shown higher response and remission rates. However, all of these methods are associated with a variable spectrum of efficacy, tolerability and safety profiles. The incidence rate of severe adverse effects commonly associated with these methods of augmentation, such as EPS and tardive dyskinesia, are found to be lower in atypical antipsychotics.

Medication should be prescribed in a personalized manner and with this in mind it is useful to note that these medicines each have a distinct profile of side effects. Drugs with sedative side effects (e.g., olanzapine and quetiapine) may not be the first choice in patients with anergic and hypersomnic symptom profiles; other patients may benefit from these properties, for example many patients with depression related sleep disturbance find the sleep promoting effects beneficial. Such a consideration of the main domains of adverse effects (e.g. extrapyramidal symptoms, weight gain, akathisia, anxiety, nausea, tremor, somnolence, sedation, hyperprolactinemia, headache, etc.) and then an attempt to prescribe in a manner which will best treat the patient’s symptoms is to be recommended. Should treatment resistant patients show inadequate response to several atypical antipsychotic augmentation trials, the addition of ECT to the ongoing antidepressant regimen may prove beneficial.
